# Exploring the psychological health of emergency dispatch centre operatives: a systematic review and narrative synthesis

**DOI:** 10.7717/peerj.3735

**Published:** 2017-10-17

**Authors:** Sarah E. Golding, Claire Horsfield, Annette Davies, Bernadette Egan, Martyn Jones, Mary Raleigh, Patricia Schofield, Allison Squires, Kath Start, Tom Quinn, Mark Cropley

**Affiliations:** 1 School of Psychology, Faculty of Health & Medical Sciences, University of Surrey, Guildford, United Kingdom; 2 School of Health Sciences, Faculty of Health & Medical Sciences, University of Surrey, Guildford, United Kingdom; 3 School of Nursing & Health Sciences, University of Dundee, Dundee, United Kingdom; 4 Faculty of Health, Social Care and Education, Anglia Ruskin University, Chelmsford, United Kingdom; 5 Rory Meyers College of Nursing, New York University, New York City, United States of America; 6 Faculty of Health, Social Care and Education, Kingston University London and St George’s, University of London, London, United Kingdom

**Keywords:** Emergency disptach, Emergency call centres, Psychological stress, Emotional stress, Job control, Systematic review, Psychological health

## Abstract

**Background:**

The study objective was to investigate and synthesize available evidence relating to the psychological health of Emergency Dispatch Centre (EDC) operatives, and to identify key stressors experienced by EDC operatives.

**Methods:**

Eight electronic databases (Embase, PubMed, Medline, CINAHL, PsycInfo, PsycArticles, The Psychology and Behavioural Sciences Collection, and Google Scholar) were searched. All study designs were included, and no date limits were set. Studies were included if they were published in English, and explored the psychological health of any EDC operatives, across fire, police, and emergency medical services. Studies were excluded if they related solely to other emergency workers, such as police officers or paramedics. Methodological quality of included studies was assessed using checklists adapted from the Critical Appraisal Skills Programme. A narrative synthesis was conducted, using thematic analysis.

**Results:**

A total of 16 articles were included in the review. Two overarching themes were identified during the narrative synthesis: ‘Organisational and Operational Factors’ and ‘Interactions with Others’. Stressors identified included being exposed to traumatic calls, lacking control over high workload, and working in under-resourced and pressured environments. Lack of support from management and providing an emotionally demanding service were additional sources of stress. Peer support and social support from friends and family were helpful in managing work-related stress.

**Discussion:**

EDC operatives experience stress as a result of their work, which appears to be related to negative psychological health outcomes. Future research should explore the long-term effects of this stress, and the potential for workplace interventions to alleviate the negative impacts on psychological health.

**PROSPERO Registration Number:**

CRD42014010806.

## Introduction

Working in a healthcare environment can be stressful; healthcare staff face a range of situational, organisational and interpersonal stressors in their roles and a significant proportion of staff report feelings of emotional exhaustion and burnout (Tuvesson, Eklund & Wann-Hansson, 2011; Gokcen et al., 2013; Andersson et al., 2016; Qiao et al., 2016). Additionally, staff in emergency settings are regularly exposed to demanding and traumatic situations, including severe injuries, aggressive individuals, and death (Lin et al., 2007; Adriaenssens, De Gucht & Maes, 2015). Emergency medical work can be unpredictable and can be physically, cognitively and emotionally demanding. Ambulance personnel have to make rapid assessments of patients and situations, and these assessments carry both professional and social responsibility. Working in stressful conditions, with repeated exposure to challenging situations, can impact negatively on both the physical and psychological health of ambulance personnel, and may lead to burnout amongst some individuals (Halpern et al., 2012; Sofianopoulos, Williams & Archer, 2012; Fjeldheim et al., 2014).

Before ambulance personnel attend a scene, the incident is first reported to an Emergency Dispatch Centre (EDC) for processing. EDC operatives do not provide any hands-on first aid; instead, they triage the incident, which involves management of a unique set of challenges. They cannot see what is occurring at the scene and are reliant upon the caller’s descriptions, which may be ambiguous, to infer the patient’s needs. EDC operatives must interrogate the caller, who may be distressed, to collect as much information as possible and assess the call’s urgency. The operative then relays relevant information about the patient’s condition and location to an ambulance crew, and if necessary, the operative remains on the line to provide immediate life-saving advice.

Although they are removed from the scene of the incident, EDC operatives work in an environment characterised by some of the same difficulties encountered by front-line responders (e.g., paramedics, police officers or fire-fighters). For example, operatives face the demands of rapid risk assessment, time-limited decision-making, and coping with unexpected developments, regardless of which emergency service they are supporting (Artman & Waern, 1999; Blandford & Wong, 2004). Additionally, EDC operatives share the issues experienced by other call-centre operatives, such as high workload, performance monitoring, and limited opportunity for physical movement (Norman et al., 2004; Snooks et al., 2008; Dean & Rainnie, 2009; Taylor et al., 2013). Stressors present in non-emergency call-centres are associated with negative physical and psychological outcomes for call-centre staff, including reduced job satisfaction, poorer work performance and increased emotional exhaustion (Norman et al., 2004; Farquharson et al., 2012; Rod & Ashill, 2013). Stress may also influence staff performance; increased stress in call-centre-based nurses has been associated with greater cognitive failures and differences in decision-making (Allan et al., 2014), and with an increased intention to leave (Farquharson et al., 2012). Finally, EDC employment also involves shift-work, which has been linked to increased health risks across a range of professions, including increased rates of cancer, obesity, cardiovascular disease, blood pressure, poorer sleep quality, burnout, depression, and anxiety (Harrington, 2001; KivimÄki et al., 2010; Wright, Bogan & Wyatt, 2013; Wisetborisut et al., 2014; Proper et al., 2016).

To date, literature reviews have focused on the sources and consequences of occupational stress that paramedics and other ambulance personnel experience (Sofianopoulos, Williams & Archer, 2012; Hegg-Deloye et al., 2014; Adriaenssens, De Gucht & Maes, 2015). In contrast, there has been no published review exploring the sources of stress that impact on psychological health when working in an EDC. Statistics from the United Kingdom show that of all staff groups across the National Health Service, ambulance service staff have the highest rate of sickness absence (Health & Social Care Information Centre, 2016), and that among emergency personnel, those working in the EDC have especially high rates of sickness absence and turnover (South East Coast Ambulance Service NHS Foundation Trust, 2015). It is therefore important to explore the stressors experienced by EDC operatives, as this knowledge could inform strategies to help minimise the impact of such stressors on the psychological health of those individuals who work in an EDC.

The objectives of this systematic review were therefore to investigate and synthesize available evidence relating to the psychological health of EDC operatives, and to identify key stressors that they experience. The specific review question was: what elements of working in the EDC do operatives identify as influencing their psychological health (e.g., reports of negative symptoms of stress, burnout, and mental health conditions, or reports suggesting strong emotional resilience and positive coping skills)?

## Methods

This review was conducted in line with the PRISMA guidelines (see Supplemental Information 2 for checklist) and was registered on the PROSPERO database (http://www.crd.york.ac.uk/PROSPERO): registration number CRD42014010806.

### Search strategy and screening

Eight electronic databases (Embase, PubMed, Medline, CINAHL, PsycInfo, PsycArticles, The Psychology and Behavioural Sciences Collection, and Google Scholar) were searched during May 2016. The contents of the Journal of Paramedic Practice were also reviewed, but this search did not uncover any additional relevant papers. Full search strings are reported in Supplemental Information 3.

Titles and abstracts of articles retrieved from databases were screened for eligiblity against the inclusion criteria (see below) to determine the population being studied, outcome measures, and study design. Any studies not excluded during abstact review were then sourced for full-text reading. The reference lists of any articles deemed eligible for inclusion after full-text reading were also reviewed, screened and potentially eligible articles retrieved. This process continued until no new articles were identified.

### Inclusion criteria for studies in the review

Inclusion criteria to determine which studies would be reviewed were developed using the PICOS acronym (Participants, Intervention, Comparator, Outcome Measures, Study Design) as recommended by The Centre for Research and Dissemination (2009). Only studies published in English were included. No date restrictions were set.

Although the primary population of interest for the review was EDC operatives who work in emergency medical settings, there was a concern that there might be insufficient evidence exploring this under-researched group. An initial literature search conducted when piloting search strings for the review confirmed this was likely to be the case. Therefore, it was deemed appropriate to include studies from other emergency services, as staff working in all these settings are likely to face similar issues, such as distressed callers, the need to risk assess each call, and time-pressured decision-making. Additionally, in some countries, EDC operatives are responsible for calls relating to more than one emergency service (e.g., the USA, Sweden).

***Participants:*** Include emergency (ambulance, fire, police) call-handlers and dispatchers (EDC operatives) working in EDCs. Exclude other emergency responders, such as paramedics, police officers, or fire-fighters.

***Intervention:*** Any intervention, where applicable.

***Comparator:*** Either another intervention, or no intervention (i.e., usual practice or care), where applicable.

***Outcome Measures:*** Any psychological health outcome in relation to working within an EDC.

***Study Design:*** Any. To include randomized controlled trials, cohort studies, cross-sectional surveys, and qualitative studies.

### Data extraction

Data extraction was completed by a team of reviewers working in pairs. Each member of a pair independently reviewed and rated their allocated papers, before agreeing final ratings. Collation of ratings and data extraction was overseen by two co-ordinating reviewers to ensure consistent application of assessment tools by all reviewers. Any discrepancies were discussed and amendments agreed.

Data extracted varied according to study design, but in all cases included information about participants and key findings. For qualitative studies, the analytic method and key themes were summarised. For quantitative studies, primary outcome measures were reported.

### Assessment of bias

The assessment tools used were adapted from the Critical Appraisal Skills Programme checklists (www.casp-uk), as there are qualitative and quantitative versions to enable appraisal across different study designs. For each study, a set of key questions were considered. If the relevant information was clearly reported, a question was scored as ‘yes’. If there was any doubt, or information was not reported in the article, that question was marked as ‘no’ or ‘can’t tell’. Studies were given an overall rating of ‘strong’, ‘moderate’, or ‘weak’, based on the number of questions scored as ‘yes’; studies had to be scored as ‘yes’ on the majority of questions to be rated as ‘strong’ overall (see Supplemental Information for details, as the criteria differed by study design). Greater emphasis was placed upon studies rated as ‘strong’ or ‘moderate’ overall within the findings of the review (Petticrew & Roberts, 2006).

### Data synthesis

Due to heterogeneity of included studies a narrative synthesis was undertaken, using a qualitative approach based on thematic analysis (Braun & Clarke, 2006). Through discussion between four reviewers, key findings from each study were extracted, coded, and grouped into themes and sub-themes. The goal of thematic analysis is to develop a set of themes that are related, but conceptually distinct, and that together illustrate important aspects of the data being analysed. Applying thematic analysis is an iterative and interpretative process, and the themes presented in this review were developed inductively from the data extracted from the included studies. Evidence from both qualitative and quantitative studies was integrated into the findings.

## Results

### Studies included in review

**Figure 1:**
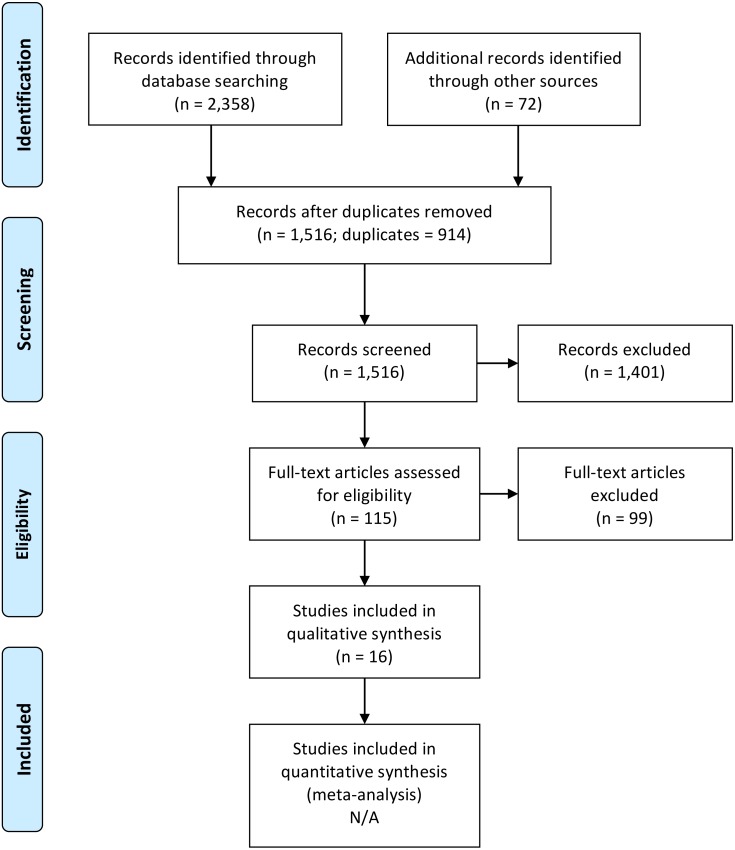
PRISMA flowchart.

A total of 2,358 articles were identified from electronic searching, of which 914 were removed as duplicates. A review of the reference lists of articles included in the review after full-text reading identified a further 72 articles that might potentially meet the inclusion criteria. Therefore, a total of 1,516 abstracts were retrieved for screening. During abstract review, 1,401 articles were excluded, leaving 115 articles to be assessed through full-text reading. After sourcing these for full-text reading, 99 articles were excluded for the following reasons: sample did not include EDC operatives (*n* = 71), findings were not related to psychological health or stress (*n* = 18), documents retrieved were unpublished articles or theses (*n* = 10). This resulted in 16 articles meeting the inclusion criteria. These details are summarised in Fig. 1.

### Study characteristics

Extracted data relating to key study characteristics are presented in Tables 1–4. The search returned seven qualitative and nine quantitative studies (six cross-sectional surveys and three cohort studies); no longitudinal studies or trials of interventions were identified.

Studies were conducted in a range of countries; seven were from the USA (Doerner, 1987; Jenkins, 1997; Tracy & Tracy, 1998; Shuler & Sypher, 2000; Pierce & Lilly, 2012; Anshel, Umscheid & Brinthaupt, 2013; Palmer, 2014), four were from the UK (James & Wright, 1991; Wastell & Newman, 1996; Sprigg, Armitage & Hollis, 2007; Coxon et al., 2016), two from Australia (Shakespeare-Finch, Rees & Armstrong, 2014; Adams, Shakespeare-Finch & Armstrong, 2015), one from France (Weibel et al., 2003), one from Sweden (Forslund, Kihlgren & Kihlgren, 2004), and one from Ireland (Gallagher & McGilloway, 2008). Populations studied included operatives across different emergency services; eight studied ambulance EDC operatives (James & Wright, 1991; Wastell & Newman, 1996; Weibel et al., 2003; Sprigg, Armitage & Hollis, 2007; Gallagher & McGilloway, 2008; Shakespeare-Finch, Rees & Armstrong, 2014; Adams, Shakespeare-Finch & Armstrong, 2015; Coxon et al., 2016), one studied fire EDC operatives (Palmer, 2014), and five studied police EDC operatives (Doerner, 1987; Tracy & Tracy, 1998; Shuler & Sypher, 2000; Pierce & Lilly, 2012; Anshel, Umscheid & Brinthaupt, 2013). In one study EDC operatives handled fire and ambulance calls (Jenkins, 1997), and in one study EDC operatives handled fire, police, and ambulance calls (Forslund, Kihlgren & Kihlgren, 2004).

**Table 1:** Study characteristics—qualitative studies.

Authors (year)	Research aim(s)	Theoretical approach	Method & data collector	Setting	Sample population	Recruitment strategy	Sample size	Inclusion/ Exclusion criteria	Analytic method
Adams, Shakespeare-Finch & Armstrong (2015)	Investigate lived experiences of EDs, with a focus on mental health and wellbeing	IPA	Face-to-face, or telephone, or Skype, semi-structured interviews; first author	Private offices (face-to-face interviews). EDs from three communications centres of one state-wide ambulance service, Australia	Ambulance EMDs	Recruited via email. After 1st nine interviews, participants strategically targeted by region, age & gender	*n* = 16, 10 women	None reported	IPA
Coxon et al. (2016)	Explore daily working experiences & how EDs manage stressors	No theoretical framework used.	Face-to-face, semi-structured interviews; first author	At worksite, in a quiet room. Single-site, ambulance EDC, South England	Ambulance EDC staff	Purposive sampling	*n* = 9, 5 women	None reported	Thematic analysis
Forslund, Kihlgren & Kihlgren (2004)	Explore situations EDs find difficult & their reflections of how they manage them	Phenomemo logical- hermeneutic approach	Face-to-face Interviews; not reported	At work-site, in a private room. Single-site, EDC, Sweden	EDC operators	Voluntary with 100% recruitment	*n* = 16, 10 women	None reported	Phenomeno logical- hermenutic approach
Gallagher & McGilloway (2008)	(1) Assess nature & impact of critical incidents on health & wellbeing, (2) Examine attitudes towards support & explore barriers	No theoretical framework used	Face-to-face Interviews; not reported	Setting of interviews not reported. Large ambulance service dispatch centre, Ireland	EMTs & EDs at ambulance EDC	Volunteers following initial survey	*n* = 27, 21 EMTs, 6 EDs, gender not reported	None reported	Thematic analysis
Palmer (2014)	Explore stressors faced by wildland fire fighting EDs & coping strategies use	Phenomenology	Face-to-face Interviews; author	Participants’ respective worksites. Multi-site, wildland fire fighter dispatch centre, USA	EDs working with wildland fire fighters	Recruited by phone & email invitation via a list provided by site co-ordinator	*n* = 11, 6 women	Inclusion criteria: at least one season of previous operational experience as a fire fighter	Phenomeno- logical
Shuler & Sypher (2000)	(1) Explore emotional labour expectations of police EDs, (2) Explore positive functions of emotional labour	Social construction of communication	Case study observation, interviews, & analysis of recorded phone calls; first author	Police EDC, in interview rooms. Single-site, Mid-western USA, mixed urban & rural	Police ED staff (*n* = 17) & the Director	Not reported	*n* = 16 EDs, gender not reported	None reported	Open-coding technique
Tracy & Tracy (1998)	(1) Explore emotional experiences of police call takers, (2) Explore the institution’s emotion expression & feeling rules, (3) Explore communicative practices used to cope during & after calls	No theoretical framework used	Case study observation, semi-structured interviews, document analysis; not reported	Single-site Police call centre. Mid-West USA	Police call takers	Not reported	*n* = 6 call takers, *n* = 1 police captain, *n* = 1 call taker trainer, gender not reported	None reported	Constant comparative method

**Notes.**

Abbreviations ED emergency dispatchers EDC emergency dispatch centre EMD emergency medical dispatchers EMT emergency medical technician IPA Interpretative Phemonenological Analysis USA United States of America

Reported sample sizes in included studies ranged from nine (Coxon et al., 2016) to 27 (Gallagher & McGilloway, 2008) individuals in the qualitative studies and eight (Weibel et al., 2003) to 358 (James & Wright, 1991) individuals in the quantitative studies. The year of study publication ranged from 1987 to 2016. One study was published in the 1980s (Doerner, 1987), four were published in the 1990s (James & Wright, 1991; Wastell & Newman, 1996; Jenkins, 1997; Tracy & Tracy, 1998), five between 2000 and 2010 (Shuler & Sypher, 2000; Weibel et al., 2003; Forslund, Kihlgren & Kihlgren, 2004; Sprigg, Armitage & Hollis, 2007; Gallagher & McGilloway, 2008), and six after 2010 (Pierce & Lilly, 2012; Anshel, Umscheid & Brinthaupt, 2013; Palmer, 2014; Shakespeare-Finch, Rees & Armstrong, 2014; Adams, Shakespeare-Finch & Armstrong, 2015; Coxon et al., 2016).

**Table 2:** Summary of findings—qualitative studies.

Authors (year)	Key themes	Summary of key findings
Adams, Shakespeare-Finch & Armstrong (2015)	(1) Operational stress and vicarious trauma, (2) Organizational stress, (3) Post traumatic growth	Dispatchers experience vicarious trauma, which can impact their mental health and relationships with others. Organizational protocols contribute to stress, & lack of positive feedback leaves dispatchers feeling undervalued. Some dispatchers experience post-traumatic growth.
Coxon et al. (2016)	(1) How dispatch is perceived by others, (2) What dispatch really involves, (3) Dealing with the stresses of dispatch	Dispatchers enjoy & take pride in work, despite stressors. Demoralisation reported. Three key stressors: resources & pay, interpersonal difficulties, & feeling overworked & undervalued.
Forslund, Kihlgren & Kihlgren (2004)	(1) Uncertainty, (2) Communication difficulties, (3) Internal & external resources, (4) Personal qualities, (5) Acquired skills	Stressors reported include: clinical uncertainty & lack of information, communication difficulties & lack of resources. Dispatchers drew on personal attributes, experience & knowledge to manage difficult calls.
Gallagher & McGilloway (2008)	(1) The nature of the critical incident(s), (2) The impact of the critical incident(s), (3) Organisational hassles, (4) The perceived effectiveness of peer support, (5) EMCs—the ‘forgotten few’?	Some critical incidents are more traumatic than others, & have more significant impact on mental health. Cumulative effect of several critical incidents occurring within a short time reported.
Palmer (2014)	(1) Stressors, (2) Coping	Themes of control & the need for emotional support to cope. Elements of organizational operations & how they contribute to worker stress were present & underscore the importance of smooth operations & supportive management in high stress jobs.
Shuler & Sypher (2000)	(1) Emotional labour as comic relief, (2) Emotional labour as ‘fix’, (3) Emotional labour as altruistic service	Emotional labour is an important aspect of the ‘structuation of organisational reality’. Healthy, positive workplaces are fostered through shared emotional labour.
Tracy & Tracy (1998)	(1) Channelling citizens’ emotion, (2) Expressed emotions of call takers, (3) Institutional feeling rules, (4) Emotion labour strategies	Call taking not considered stressful of itself. Peer-support helps reduce stress. Advantage of telephone versus face to face exchanges where emotional demand high. Organisational culture influences emotional expression.

**Table 3:** Study characteristics—quantitative studies.

Authors (year)	Research aim(s)	Setting, role, region & country	Gender & Age (yrs)	Ethnicity	Education	Av. exp. (yrs)	Recruitment methods	Inclusion/Exclusion criteria	Is sample deemed representative of population?
Anshel, Umscheid & Brinthaupt (2013)	Determine effects of 10-week coping skills & fitness training programme on changes in perceived stress, job satisfaction, and perceived energy.	Police dispatch centre. Medium-sized city, South Eastern USA.	89% female; *rge*= 24–45; *m* = 35	NR	NR	8	Volunteers recruited after oral presentation about the study	Full-time ED	No check on representativeness reported
Doerner (1987)	Examine levels of stress in police dispatchers in a municipal law enforcement agency	Police dispatch centre (21 dispatchers, five supervisors, five community service officers). Urban, Tallahassee, Florida, USA	NR; NR	NR	NR	NR	Study materials distributed at start of each shift	Total cohort	No check on representativeness reported
James & Wright (1991)	Identify types of situation typically identified as stressful by ambulance service staff	Devon Ambulance Service; ambulance crew, officers & control room staff. Urban & rural South West UK	NR; NR	NR	NR	NR	Survey posted to all employees	Total cohort	Total cohort sample, no check on representativeness reported
Jenkins (1997)	Examine relationships between acute disaster stress, coping, social support networks & physical, psychological stress in emergency dispatchers following exposure to a hurricane	Dade County Communications Centre (fire & ambulance dispatch), South Florida, USA	93% female; *rge*= 23–59; *m* = 38	7% African American, 62% Caucasian, 24% Hispanic, 6% Native American	35% high school, 33% technical/ college education	12	Volunteers from two out of three shifts; not total cohort	NR	No check on representativeness reported, but no participants from one shift
Pierce & Lilly (2012)	Explore work-related trauma exposure, peri-traumatic distress & PTSD symptomatology	Police tele-communicators, 24 states, Midwestern & Southwestern USA	74% female; *m* = 39	77% Caucasian	81% min. college education or vocational training	12	Convenience sample recruited via letters, adverts to professional associations, online forums, social media outlets	Inclusion: at least part-time work in the last year as a tele-communicator. No exclusion criteria applied	No check on representativeness reported
Shakespeare-Finch, Rees & Armstrong (2014)	Investigate the impact of self-efficacy, giving & receiving social support on psychological well-being, post-traumatic growth & symptoms of PTSD	EMDs, three different regions across Queensland, Australia	68% female; *n* = 20 aged >45, *n* = 34 aged 26–45, *n* = 6 aged 22–25	NR	NR	NR	Managers of communication centres sent group emails to all employees	Only inclusion criteria: employed as EMD	No check on representativeness reported
Sprigg, Armitage & Hollis (2007)	Investigate prevalence, perceived causes of verbally abusive calls, & relationship to psychological wellbeing.	NHS ASCR, UK, region not reported	81% female; *m* = 33	98% Caucasian, 2% Other	NR	NR	Recruited from ASCR, but further details not reported	NR	Can’t tell as recruitment methods not clear.
Wastell & Newman (1996)	Evaluate the psychophysiological impact of new computerised ‘ALERT’ control system by comparing job performance & stress before & after implementation	Ambulance control room. Urban, Manchester, UK	90% female; *rge* = 19-55	NR	NR	NR	NR	NR	Can’t tell as recruitment methods not clear.
Weibel et al. (2003)	Establish diurnal salivary cortisol levels in EMD centre.	EMD Centre. Urban, Metz, France	88% female; *rge*= 36–53	NR	Reported as variable	4	NR	Inclusion (EMD group): working in ED >1 yr; working on same rotation; participation on shift following rest period of 7 days. Inclusion (control group, not EMD staff): lab staff on rest days. Exclusion (all): Not on any medication, good general health, no history of psychiatric illness.	Can’t tell as recruitment methods not clear & sample size of 8 in each group.

**Notes.**

Abbreviations ASCR ambulance service control room Av. Exp. average experience ED emergency dispatch EMDs emergency medical dispatchers m mean NHS National Health Service NR not reported PTSD post-traumatic stress disorder *rge* range UK United Kingdom USA United States of America

**Table 4:** Summary of findings—quantitative studies.

Authors (year)	Cohort studies: sample size	Surveys: mode of delivery, sample size & response rate	Enough statistical power?	Outcome variable (measure)	Follow-up	Confounders controlled?	Results	Summary
Anshel, Umscheid & Brinthaupt (2013)	EDs *n* = 9	N/A	No	Perceived stress (PSS), job satisfaction (JS), perceived physical energy (13 item measure created by authors), coping style for acute stress (15 item measure created by authors), fitness testing (upper and lower body muscular strength)	Pre-post study	No control for possible confounders	All participants provided with 12-week membership to local fitness club & assigned personal performance coach. Upper & lower body strength & perceived physical energy significantly higher at post-test. No increase in approach-coping strategies; increase in avoidance-coping strategies, but not statistically significant. Perceived stress was lower at post-test, close to statistical significance. No difference in job satisfaction over time.	The coping skills & fitness programme increased perceived physical energy & body strength, & reduced perceived stress, but had no effect on coping strategies or job satisfaction.
Doerner (1987)	N/A	31 distributed, *n* = 22. EDs & supervisors 69% response, community service officers 80% response. Distributed at start of each shift	No	State anxiety & trait anxiety (STAI)	None	No control for possible confounders	Male EDs had greater TA & SA than community norms. Female EDs had greater TA than community norms. Most-liked aspects (frequency-based questions): helping people, assisting officers, responding to telephone inquiries, excitement & adventure involved in radio work, variety on the job. Negative aspects (frequency-based questions): understaffing & overload, public disrespect & rudeness	Police EDs show elevated stress scores, but are not pathologically stressed. Police EDs report job satisfaction from the range of tasks they perform, but face organisational barriers that affect efficient working
James & Wright (1991)	N/A	Distributed to 518 employees, 358 returned (69% response rate), postal	Yes	Common stressors identifier (42 item measure, developed by authors)	None	N/A	Four factor structure of common stressors identified (1) Organisational & managerial aspects; (2) New, unfamiliar & difficult duties/uncertainty; (3) Work overload; (4) Interpersonal relations	Key stressful situations include dealing with injured children, underuse of ability & potential, inadequate pay, managerial support & facilities at work. How ambulance staff are treated by other colleagues is an area of severe stress.
Jenkins (1997)	N/A	Total number distributed not reported. *n* = 68, materials hand delivered to participants who completed at work & returned by post	No	Social support (IQ), acute stress (ADSI) coping with effects of storm (WOC), effects of stress (IES), worst health symptom (health survey, author) general psychological distress (BSI GSI)	None	Completer analysis reported; no control for demographic or other confounders	Mean IES scores comparable to other disaster worker studies for Intrusion & for Avoidance. SS negatively associated with distress. EDs who were at work during storm were not more distressed at 2.5 mths than those who were at home. EDs who received CISD were higher in avoidance at 2.5 mths, but they also had higher estimated uninsured property losses & less social network involvement	Effects of storm on EDs were comparable to other disaster workers. CISD associated with higher avoidance, but cannot rule out effect of property damage. Social support deficits associated with greater distress
Pierce & Lilly (2012)	N/A	Total distributed not reported. *n* = 171 police tele-communicators, option for either hard-copy or online completion	Yes	Exposure to potentially traumatic events/calls (PTEM). Emotional distress related to worst duty-related event (PDI). PTSD symptoms over last 1 mth (PDS)	None	Demographic variables not checked or controlled for	Participants reported experiencing fear, helplessness or horror in reaction to 32% of different types of calls. Peri-traumatic distress: average score was 2.93, higher than comparator sample of police officers (1.3) & civilians (1.69) (comparator sample from Brunet et al., 2001). PTSD symptoms: 3.5% scored at or above 28 (cut-off for probable, current PTSD). Significant positive correlation between peri-traumatic distress & PTSD symptoms	Peri-traumatic distress was high & positively correlated with PTSD symptoms. Despite tele-communicators being physically distinct from traumatic scene, authors suggest they may not be buffered from development of PTSD symptoms
Shakespeare-Finch, Rees & Armstrong (2014)	N/A	Online survey. 120 surveys distributed. *n* = 60 (50% response rate). Only 44 participants reported experiencing a trauma, & were treated as a ‘trauma sub-sample’	No	Wellbeing (PWS), post-traumatic stress symptomatology (IES-R) & PTG (PTGI). Possible predictors self-efficacy (NGES), shift-work, being a trained ‘peer support officer’, giving & receiving SS (2-SSS)	None	Demographic variables not checked or controlled for	All EMDs reported high levels of self efficacy, total SS & the giving of SS. Self-efficacy, giving SS & receiving SS positively predicted psychological wellbeing across main sample (*n* = 60). 44 participants reported experiencing trauma; these 44 EMDs only were examined for predictors of PTSD symptoms & PTG. Shift work & receiving SS negatively predicted PTSD symptoms in trauma subsample. Receiving SS positively predicted PTG in trauma subsample	Self-efficacy & receiving SS positively associated with psychological wellbeing. Receiving SS also related to higher levels of PTG & lower levels of PTSD
Sprigg, Armitage & Hollis (2007)	N/A	Total distributed not reported. *n* = 48, survey method of delivery not reported	No	No. of verbally abusive calls, emotional exhaustion, health (GHQ), job-related strain & employee turnover intent (other measures not named). Perceived perpetrators of & perceived reasons for verbal abuse	None	Demographic variables not checked or controlled for	7% of calls on average verbally abusive. Patients & emergency callers perceived as greater source of abuse than other professionals. ASCR staff perceived caller frustration & anxiety as most common cause of verbal abuse. Greater no. of abusive calls significantly positively correlated with levels of emotional exhaustion, depersonalisation & anxiety, but not depression & GHQ scores. Organisational commitment not significantly correlated with no. of calls, but significantly negatively correlated with all other outcomes	Call handlers reporting greater no. of abusive calls reported higher levels of emotional exhaustion, depersonalisation, & anxiety, but not depression & GHQ scores. Organisational commitment lower in those experiencing physical & psychological distress
Wastell & Newman (1996)	Total staff *n* = 45, but not clear if all took part	N/A	Unclear	External work demands (counted no. of simultaneously active jobs handled by ED at time of sampling). HR, BP, anxiety & fatigue (VAS), ED perceptions of ALERT after implementation	Before & after study; approx. 4 mths after implementation of ALERT	Not reported	Improvements in performance with ALERT; ambulances arriving within 8 mins increased from 55.4% to 64.4%. No significant difference between HR & BP between paper based & computer based system, but increased workload demands associated with increase in BP in paper system only. Anxiety & fatigue increased with workload in both systems, but effect greater in paper system. EDs reported reduced stress levels & improvement in service performance using ALERT	Perceived stress reduced & service performance improved following implementation of ALERT
Weibel et al., 2003	EMDs *n* = 8; controls *n* = 8. Controls were staff in author’s lab, matched for age, sex & smoking status	N/A	No	Salivary cortisol (sampled every 2hrs, from 9am to 7pm, across 1 day), subjective stress perception, attitude toward work (measures not named)	None	Not reported	Daytime cortisol secretion higher in EMDs than controls. Both groups: cortisol levels decreased towards end of day. Cortisol levels constantly higher in EMDs. Positive correlation between individual total cortisol levels & perceived emotional stress. Poor physical work environment positively associated with poor relationships with hierarchy	EMDs experience higher secretion of cortisol levels compared to lab staff on rest days

**Notes.**

Abbreviations ADSI Acute Disaster Stress Index ASCR ambulance service control room approx. approximately BP blood pressure BSI GSI Derogatis Brief Symptom Inventory Global Severity Index CISD Critical Incident Stress Debriefing ED emergency dispatcher GHQ General Health Questionnaire HR heart rate IES Impact of Events Scale IES-R Impact of Events Scale-Revised IQ Incident Questionnaire JS general job satisfaction measure mins minutes mth month NGES New General Efficacy Scale no. number PDI Peri-traumatic Distress Inventory PDS Post-traumatic Diagnostic Scale PSS Perceived Stress Scale PTEM Potentially Traumatic Events/calls Measure PTG post-traumatic growth PTGI Post Traumatic Growth Inventory PTSD post-traumatic stress disorder PWS Psychological Wellbeing Scale SA state anxiety SS social support STAI State-Trait Anxiety Inventory TA trait anxiety VAS visual analogue scales WOC Ways of Coping Questionnaire 2-SSS 2-way Social Support Scale

**Table 5:** Study assessments—qualitative studies.

Authors (year)	Aims clearly stated?	Qualitative method appropriate?	Design appropriate to address aims?	Recruitment strategy appropriate?	Data collected so as to address research issue?	Relationship between researcher & participants adequately considered?	Ethical issues considered?	Data analysis sufficiently rigorous?	Findings clearly stated?	Overall quality rating
Adams, Shakespeare-Finch & Armstrong (2015)	Yes	Yes	Yes	Yes	Yes	No	Yes	Yes	Yes	Strong
Coxon et al. (2016)	Yes	Yes	Yes	Yes	Yes	No	Yes	Yes	Yes	Strong
Forslund, Kihlgren & Kihlgren (2004)	Yes	Yes	Can’t Tell	Yes	Yes	No	No	No	No	Weak
Gallagher & McGilloway (2008)	Yes	Yes	Yes	Can’t tell	Yes	No	Can’t tell	No	Yes	Moderate
Palmer (2014)	Yes	Yes	Yes	Yes	Yes	No	Yes	Can’t Tell	Yes	Moderate
Shuler & Sypher (2000)	Yes	Yes	Yes	Can’t tell	Yes	No	Can’t tell	Yes	Yes	Moderate
Tracy & Tracy (1998)	Yes	Yes	Yes	Can’t tell	Yes	Can’t tell	Can’t tell	Yes	Yes	Moderate

**Table 6:** Study assessments—quantitative studies.

Authors (year)	Did study address a clearly focused issue?	Cohort/ sample recruited in an acceptable way?	Exposure accurately measured to minimise bias?	Outcome accurately measured to minimise bias?	Have authors identified important confounding factors?	Have authors accounted for confounding factors in design and/or analysis?	Was follow up of participants long enough?	Do you believe the results?	Can results be more widely applied?	Overall quality rating
Anshel, Umscheid & Brinthaupt (2013)	Yes	Yes	Can’t tell	Yes	No	No	Can’t tell	Can’t tell	Can’t tell	Weak
Doerner (1987)	Yes	Yes	N/A	No	N/A	N/A	N/A	No	No	Weak
James & Wright (1991)	Yes	Yes	N/A	Can’t tell	N/A	N/A	N/A	Can’t tell	Can’t tell	Weak
Jenkins (1997)	Yes	Can’t tell	N/A	Yes	N/A	N/A	N/A	Yes	Can’t tell	Moderate
Pierce & Lilly (2012)	Yes	Yes	N/A	Yes	N/A	N/A	N/A	Yes	Yes	Strong
Shakespeare-Finch, Rees & Armstrong (2014)	Yes	Yes	N/A	Yes	N/A	N/A	N/A	Can’t tell	Can’t tell	Moderate
Sprigg, Armitage & Hollis (2007)	Yes	Can’t tell	N/A	Can’t tell	N/A	N/A	N/A	Yes	Can’t tell	Weak
Wastell & Newman (1996)	Yes	Yes	Yes	Yes	Can’t tell	Can’t tell	Can’t tell	Yes	Yes	Moderate
Weibel et al. (2003)	Yes	Can’t tell	Can’t tell	Yes	Yes	No	No	No	Can’t tell	Weak

### Risk of bias within studies

Details of the application of the assessment tool are shown in Tables 5 and 6. One qualitative (Forslund, Kihlgren & Kihlgren, 2004) and five quantitative (Doerner, 1987; James & Wright, 1991; Weibel et al., 2003; Sprigg, Armitage & Hollis, 2007; Anshel, Umscheid & Brinthaupt, 2013) studies were assessed as ‘weak’, and were therefore deemed to be at higher risk of potential bias. Four qualitative (Tracy & Tracy, 1998; Shuler & Sypher, 2000; Gallagher & McGilloway, 2008; Palmer, 2014) and three quantitative studies (Wastell & Newman, 1996; Jenkins, 1997; Shakespeare-Finch, Rees & Armstrong, 2014) were assessed as ‘moderate’ and two qualitative (Adams, Shakespeare-Finch & Armstrong, 2015; Coxon et al., 2016) and one quantitative (Pierce & Lilly, 2012) studies were assessed as ‘strong’. The narrative results that follow are based predominantly on the findings from the ten studies judged to be either ‘moderate’ or ‘strong’.

Participant demographics were described in most studies; however, amongst the quantitative studies recruitment methods were not always clearly reported (Wastell & Newman, 1996; Weibel et al., 2003; Sprigg, Armitage & Hollis, 2007), and in one study participants were only recruited from two out of three shifts (Jenkins, 1997).

### Narrative synthesis

Evidence from both qualitative and quantitative studies were synthesised into two main themes: ‘Organisational and Operational Factors’ and ‘Interactions with Others’. Subthemes explored factors that influenced perceived levels of stress, emotional distress and/or wellbeing.

#### Theme 1: organisational and operational factors

Three subthemes were identified that related to key stressors associated with the workplace environment.

##### 1a: perceptions of control.

EDC operatives across all emergency settings generally reported a lack of control over their workload, and a lack of organisational recognition for the demands of managing their workload. Multitasking and balancing competing demands is a common feature of the role (Doerner, 1987; Palmer, 2014; Adams, Shakespeare-Finch & Armstrong, 2015; Coxon et al., 2016), but workload planning was reported as difficult due to unpredictability in the volume of calls (Jenkins, 1997). Operatives’ lack of control was exacerbated by excess workloads, role conflict, being monitored, and by a lack of understanding of their role by outsiders (Doerner, 1987; Shuler & Sypher, 2000; Palmer, 2014; Adams, Shakespeare-Finch & Armstrong, 2015; Coxon et al., 2016), which also manifested in feelings of being undervalued and demoralised.

Physical layout and constricted or inadequate workspaces added to this sense of low control and increased stress (James & Wright, 1991; Weibel et al., 2003; Palmer, 2014; Coxon et al., 2016). Ambulance operatives reported feeling out of control over their workload and current activity within their area of responsibility after returning from rest breaks; this fear of losing control meant they often chose not to take their allocated break time, even though such breaks were allowed during the working day (Coxon et al., 2016).

A lack of control over outcomes was identified as another source of stress across several studies. Police operatives sought to overcome feelings of powerlessness and gain some control through advice giving and by upping call priorities; operatives sometimes over-rode preassigned priority codes in the system to give a call an increased priority (Tracy & Tracy, 1998). Despite this, both police and ambulance operatives still sometimes felt powerless, as they had little control over, and knowledge about, outcomes for the patient or caller. If a poor outcome was reported, operatives sometimes felt responsible, especially if they felt they had been unable to influence the caller to do what was required (Tracy & Tracy, 1998; Adams, Shakespeare-Finch & Armstrong, 2015). Compounding this, operatives who had previous field experience as fire-fighters or paramedics, were further frustrated by the lack of control they had over outcomes; in the EDC they felt there was little they could do, compared to the more active roles they used to take in responding to emergencies (Palmer, 2014). Feelings of powerlessness and lack of control appeared to contribute to the stress experienced by these operatives (Tracy & Tracy, 1998; Palmer, 2014; Adams, Shakespeare-Finch & Armstrong, 2015).

More positively, in one study, self-efficacy was highlighted as being important in helping reduce stress. Operatives who believed they were capable of coping, felt better able to deal with stressors, and experienced greater wellbeing (Shakespeare-Finch, Rees & Armstrong, 2014).

##### 1b: exposure to traumatic and abusive calls.

Psychological stress associated with taking traumatic and sometimes abusive calls was explored in several studies. Despite not being physically exposed to the emergency situation, there was some evidence that EDC operatives experience the trauma vicariously. In one study, participants reported experiencing fear, helplessness, or horror in reaction to 32% of the different types of calls (e.g., calls about children, suicide or domestic violence) that they received (Pierce & Lilly, 2012). Operatives reported higher peri-traumatic distress compared to police officers, and this level of distress experienced during and immediately after a traumatic call positively correlated with post-traumatic stress disorder (PTSD) symptoms and burnout (Pierce & Lilly, 2012). PTSD symptomology was identified in police and ambulance operatives (Pierce & Lilly, 2012; Shakespeare-Finch, Rees & Armstrong, 2014; Adams, Shakespeare-Finch & Armstrong, 2015). Calls relating to road traffic accidents, incidents involving children or vulnerable adults, domestic violence, and suicides were commonly cited as the most distressing types of call (James & Wright, 1991; Tracy & Tracy, 1998; Shuler & Sypher, 2000; Forslund, Kihlgren & Kihlgren, 2004; Gallagher & McGilloway, 2008; Pierce & Lilly, 2012).

Handling traumatic calls was related to lower organisational commitment, a higher desire to leave, and greater emotional exhaustion (Sprigg, Armitage & Hollis, 2007). Exposure to potentially traumatic calls also impacted ambulance operatives’ mental health; some operatives reported angry outbursts, difficulties sleeping, nightmares, flashbacks, and increases in alcohol consumption (Gallagher & McGilloway, 2008; Adams, Shakespeare-Finch & Armstrong, 2015). Four ambulance operatives in one study reported taking long-term sick-leave in the previous year due to job-stress (Gallagher & McGilloway, 2008). Police operatives reported using both approach and avoidance coping strategies when engaging with callers (Anshel, Umscheid & Brinthaupt, 2013).

In addition to traumatic calls, some studies reported instances of abusive calls. Abuse came mostly from callers (James & Wright, 1991; Tracy & Tracy, 1998; Sprigg, Armitage & Hollis, 2007), but sometimes from fellow professionals (Sprigg, Armitage & Hollis, 2007; Gallagher & McGilloway, 2008). Operatives had to suppress their emotional reactions when dealing with these callers (Tracy & Tracy, 1998). Ambulance operatives who received more calls that were abusive were higher in emotional exhaustion, depersonalisation, and anxiety; these operatives also had a higher intention to quit their job (Sprigg, Armitage & Hollis, 2007).

Nonetheless, not all operatives experienced difficult calls negatively; in one study, participants reported gaining ‘emotional competence’ following repeated exposure to traumatic calls, and they gained additional satisfaction from the altruistic nature of their supportive role (Shuler & Sypher, 2000). Another study explored the nature of post-traumatic growth amongst ambulance operatives; participants reported finding value and a new appreciation of life as a result of their job role (Adams, Shakespeare-Finch & Armstrong, 2015).

##### 1c: making decisions in uncertain, under-resourced, and pressured environments.

A lack of high quality training was identified by some operatives as contributing to stress levels, and created a negative perception of the organisation (Doerner, 1987; Gallagher & McGilloway, 2008; Palmer, 2014; Adams, Shakespeare-Finch & Armstrong, 2015; Coxon et al., 2016). Poor training also resulted in lower personal responsibility for performance amongst ambulance operatives (Coxon et al., 2016). Untrained supervisors and a high turnover of staff created additional stress amongst fire and police operatives, due to an increased workload (Doerner, 1987; Palmer, 2014). EDC operatives in Sweden, who deal with all emergency services, emphasised limited resources in the healthcare system, which made prioritising and managing calls more challenging (Forslund, Kihlgren & Kihlgren, 2004).

The process of gathering relevant and sufficient information in an EDC is a time critical task, which in itself appears to be stressful. One study that examined the introduction of an electronic dispatching system, found the new system improved the efficiency of information gathering, compared to the previous paper-based system, and self-reported stress levels were reduced as a result (Wastell & Newman, 1996). System efficiencies, however, do not necessarily reduce stress from clinical uncertainty, resulting from distressed callers who provide insufficient information, and report vague symptoms (James & Wright, 1991; Tracy & Tracy, 1998; Forslund, Kihlgren & Kihlgren, 2004; Adams, Shakespeare-Finch & Armstrong, 2015). Operatives also experienced frustration with callers’ physical and linguistic capabilities, and those who did not fully answer questions or follow instructions, which were further barriers to their understanding of the situation (Tracy & Tracy, 1998; Forslund, Kihlgren & Kihlgren, 2004; Gallagher & McGilloway, 2008; Adams, Shakespeare-Finch & Armstrong, 2015).

Despite these challenges, some operatives appeared to enjoy the fast-paced and sometimes exciting environment of the EDC (Shuler & Sypher, 2000; Palmer, 2014; Coxon et al., 2016). The adrenaline rush and variety experienced was cited as a reason for continuing in the role (Doerner, 1987; Shuler & Sypher, 2000). Nonetheless, the demands should not be underestimated, as operatives described having to juggle several competing and equally important tasks at once, and high, relentless workloads were commonly reported (James & Wright, 1991; Gallagher & McGilloway, 2008; Palmer, 2014; Adams, Shakespeare-Finch & Armstrong, 2015; Coxon et al., 2016). Police operatives were particularly concerned about their performance in handling fluid situations, such as robberies-in-progress or suicidal callers, in case they did not make the correct decisions (Tracy & Tracy, 1998).

#### Theme 2: interactions with others

Three subthemes identified related to how operatives’ experiences of stress were influenced by their interactions with other people.

##### 2a: perceived quality of supervisory relationships.

Poor leadership frustrated EDC operatives (Gallagher & McGilloway, 2008; Palmer, 2014) and a lack of appropriate supervision enhanced the burden of professional responsibility (Palmer, 2014). A perceived lack of support, empathy, and understanding from supervisors and managers increased reported stress (James & Wright, 1991; Gallagher & McGilloway, 2008; Palmer, 2014; Coxon et al., 2016). Management were perceived as being too distant from the role to truly understand its demands (Gallagher & McGilloway, 2008), whilst in one study supervisors were reported as actively discouraging emotional expression (Shuler & Sypher, 2000). Additionally, some operatives felt that a healthy work-life balance was not always encouraged by supervisors (Palmer, 2014).

Operatives also perceived failures by management to tackle conflict amongst colleagues. Reference was made to difficulties and misunderstandings resulting from a perceived dispatcher-paramedic divide (Adams, Shakespeare-Finch & Armstrong, 2015; Coxon et al., 2016), and the seemingly invisible nature of the role (Gallagher & McGilloway, 2008). Making an effort to improve relationships with other colleagues was perceived as reducing some of this tension, although supervisors did not generally facilitate this relationship-building activity (Coxon et al., 2016). Ambulance operatives reported that bullying and inappropriate behaviour by colleagues was not properly addressed by management, causing an additional strain (Gallagher & McGilloway, 2008).

##### 2b: seeking social support from peers, friends and family.

Peer support was commonly regarded as necessary to reduce the emotional burden of the work of EDC operatives. This was the case for both informal (Tracy & Tracy, 1998; Shuler & Sypher, 2000; Palmer, 2014; Shakespeare-Finch, Rees & Armstrong, 2014; Adams, Shakespeare-Finch & Armstrong, 2015; Coxon et al., 2016) and more formal forms of peer support, such as having trained peer support officers available (Shakespeare-Finch, Rees & Armstrong, 2014). There appeared to be beneficial value from engaging in storytelling with colleagues; sharing anecdotes about callers allowed police operatives to vent frustrations, and occasionally provided comic relief (Tracy & Tracy, 1998; Shuler & Sypher, 2000). The use of black humour between peers was also mentioned by ambulance operatives (Adams, Shakespeare-Finch & Armstrong, 2015). One study found no effect on stress and coping amongst those operatives who received critical incident stress debriefing with peers, compared to those who did not (Jenkins, 1997). Another study found that staff were cynical about a formal peer support service, doubting the value and confidentiality of the service (Gallagher & McGilloway, 2008), whilst poor communication with co-workers was an additional stressor (Coxon et al., 2016).

Work-life balance, such as adequate time off and outside interests, was considered vital, as was support from family and friends (Palmer, 2014; Shakespeare-Finch, Rees & Armstrong, 2014; Adams, Shakespeare-Finch & Armstrong, 2015). Fire and police operatives who reported less social support experienced greater psychological distress following exposure to a hurricane (Jenkins, 1997), whilst having adequate social support was associated with reduced stress levels and better psychological health across all services (Jenkins, 1997; Shakespeare-Finch, Rees & Armstrong, 2014; Adams, Shakespeare-Finch & Armstrong, 2015). Not all operatives, however, reported gaining benefits from social support. Some reported feeling that friends and family lacked understanding, and so operatives sometimes chose not to discuss their job with them (Coxon et al., 2016). Ambulance operatives reported that job stress and long working hours affected their personal lives (Gallagher & McGilloway, 2008), and fire operatives reported that technology such as mobile phones made it hard to get adequate distance from the role (Palmer, 2014).

##### 2c: providing an emotionally demanding public service.

EDC operatives experienced stronger emotional reactions to certain calls, particularly those related to children, suicides, and premature deaths (Tracy & Tracy, 1998; Forslund, Kihlgren & Kihlgren, 2004; Gallagher & McGilloway, 2008). Dealing with inappropriate callers was also frustrating for operatives (Tracy & Tracy, 1998). The nature of the role, however, requires EDC operatives to remain calm and in control of their emotions when interacting with the public (Tracy & Tracy, 1998; Shuler & Sypher, 2000). Operatives reported that maintaining emotional neutrality and not expressing anger and frustration is vital for managing calls effectively, and operatives have to absorb the caller’s frustrations and anxieties (Doerner, 1987; Jenkins, 1997; Tracy & Tracy, 1998; Shuler & Sypher, 2000; Sprigg, Armitage & Hollis, 2007; Coxon et al., 2016). Maintaining this emotional neutrality can be challenging, especially when operatives self-identified with the situation (Forslund, Kihlgren & Kihlgren, 2004; Adams, Shakespeare-Finch & Armstrong, 2015).

Operatives reported that sharing emotional experiences with peers could bring benefits, especially through the use of humour and storytelling (James & Wright, 1991; Tracy & Tracy, 1998; Shuler & Sypher, 2000; Adams, Shakespeare-Finch & Armstrong, 2015). However, organisational culture sometimes resulted in a lack of opportunities in which to express emotions (Tracy & Tracy, 1998; Shuler & Sypher, 2000; Coxon et al., 2016), and operatives did not always share their emotions with colleagues (Tracy & Tracy, 1998; Shuler & Sypher, 2000).

Despite the obvious importance of the role, EDC operatives reported feeling invisible, and not fully recognised for their work, by the public, by their family, and sometimes by their colleagues, which was another source of stress (James & Wright, 1991; Gallagher & McGilloway, 2008; Coxon et al., 2016). This feeling was common across different emergency services, as well as different countries. Nonetheless, operatives did gain some benefit from feeling that they were playing an altruistic role in their service provision (Doerner, 1987; Shuler & Sypher, 2000; Palmer, 2014).

### Potential bias in the review

The findings of the narrative review were inevitably limited by the design of the studies included. Although common themes were identified across different study designs, the review highlighted a lack of longitudinal and experimental research. Thus, conclusions drawn about the relationships between EDC-related stressors and the psychological health of EDC operatives may be limited by the methodological approaches employed in the original studies.

## Discussion

Research examining Emergency Dispatch Centre (EDC) operatives’ psychological health is limited. This narrative review identified that EDC operatives across different emergency services consistently reported their job as highly stressful, and this stress sometimes affected their psychological health. It also highlighted a lack of longitudinal studies exploring the long-term effects on psychological health of working in an EDC.

Workload demands and supervisory difficulties were frequent concerns. Although EDC operatives do receive supervision and training, some operatives considered their training and supervision to be inadequate, which they reported exacerbated their stress. Poor supervision, a lack of understanding by management, and the pressures of being monitored have also been identified as potential stressors amongst staff in other call-centre and medical settings (Deery, Iverson & Walsh, 2002; Dean & Rainnie, 2009; Taylor et al., 2013; Johansen, 2014).

Frustrations arose amongst EDC operatives from a perceived lack of control over their workload, and this might have implications for their wellbeing and retention, as perceptions of job control amongst non-emergency call-centre agents are related to job satisfaction (Grebner et al., 2003). Furthermore, an excessive and demanding workload is a common feature across call-centre work, and this may lead to emotional exhaustion and burnout (Deery, Iverson & Walsh, 2002; Dean & Rainnie, 2009; Rod & Ashill, 2013; Taylor et al., 2013).

The nature of the calls is another source of considerable stress. Although EDC operatives do not experience emergencies first hand, operatives can experience vicarious trauma as a result of some of the calls they handle. Research exploring the effects of job-related exposure to trauma in emergency department personnel (Lin et al., 2007; Adriaenssens, De Gucht & Maes, 2015) and trainee paramedics (Fjeldheim et al., 2014) suggests that such exposure can have negative impacts on health and wellbeing, and may contribute to burnout (Adriaenssens, De Gucht & Maes, 2015) and compassion fatigue (Van Mol et al., 2015). It seems reasonable to assume that EDC operatives are at risk of similar outcomes. The findings from this review indicate that some EDC operatives do indeed experience symptoms associated with negative psychological health and high levels of psychological stress, including increased alcohol use, disturbed sleep, and unwanted flashbacks related to calls. Similar symptoms, along with high rates of depression and post-traumatic stress disorder symptomology, have been reported in both trainee (Fjeldheim et al., 2014) and qualified (Hegg-Deloye et al., 2014) paramedics.

EDC operatives are therefore likely to be at risk of emotional exhaustion and burnout, which may contribute to high absence rates seen amongst this group (South East Coast Ambulance Service NHS Foundation Trust, 2015). Some EDC operatives experience conflict and abuse from callers, which could further contribute to burnout. Call handlers working for a telecommunications company who believed customers were more abusive experienced greater emotional exhaustion (Deery, Iverson & Walsh, 2002). Additionally, those who reported higher levels of emotional exhaustion also had higher rates of absence (Deery, Iverson & Walsh, 2002). Burnout rates in other emergency medical personnel are also high (Lin et al., 2007; Popa et al., 2010; Adriaenssens, De Gucht & Maes, 2015), although this can vary by context (Popa et al., 2010; Gokcen et al., 2013). Educational programmes may help to reduce compassion fatigue and burnout (Flarity, Gentry & Mesnikoff, 2013).

Some EDC operatives reported using strategies to mitigate some of the negative effects of workplace stressors; strategies included the use of emotion regulation and engaging in peer support. Some of the evidence from this review, however, suggests the EDC work environment does not always provide opportunities for sufficient peer support. Existing research from other emergency medical settings suggests that the peer support element of team working can be beneficial for managing stress and preventing burnout (Beaton et al., 1997; Halpern et al., 2012). Future interventions may therefore need to focus on increasing opportunities for peer support, to build resilience amongst EDC operatives.

It is clear from the review that working as an EDC operative is highly demanding, and that stress will be inevitable at times. Interventions in the form of cognitive behaviour therapy or mindfulness (Hülsheger et al., 2014; Querstret et al., 2016; Querstret, Cropley & Fife-Schaw, 2017) have been shown to reduce potential negative effects of working in highly demanding environments. Future research could explore how similar interventions might help EDC staff to cope effectively with work-related stress.

It should also be noted that despite the challenges of the role, and the potential risks for psychological health, the studies reviewed did identify some of the more positive aspects of the role. EDC operatives enjoy performing an altruistic role, and reported gaining emotional competence as they gained experience, with some operatives also experiencing post-traumatic growth. The EDC work environment is fast-paced, demanding, and varied, which operatives saw as a key reason they enjoy their work and remain in the role. Indeed, exploring why some operatives experience higher levels of role-related benefits and fewer negative effects on their psychological health might indicate potential areas to intervene with their colleagues who do not have such positive experiences.

The findings from this review therefore highlight the unique combination of challenges faced by EDC operatives. The role combines features of both regular call-centres and emergency settings. Operatives report facing a psychologically demanding work environment, and at times, the workload can seem relentless. Although some operatives appear to thrive on the challenge, a significant proportion of the EDC workforce report experiencing negative effects on their psychological health as a result of their work. Future research should focus on exploring whether workplace-based interventions can improve the psychological health of those EDC operatives who are negatively affected.

## Limitations

Search strings were devised to capture a broad range of studies, and a number of common themes were identified. The diversity of EDC populations studied (across time, role, and location) means that the relative importance of each theme is likely to vary between each sub-population of EDC operatives; there were, however, insufficient publications to enable differences between the emergency services to be explored. As there is a lack of recent evidence in particular, the findings of the review are potentially both time- and context-bound. Results should be interpreted with caution as findings are based on a limited number of high quality studies and there was heterogeneity in study design. Nonetheless, there was evidence of consistency across studies, as the themes identified were supported by evidence from all three emergency services, suggesting that the findings of the review are likely to be relevant to all types of EDC operatives.

The review was intended to capture longitudinal and intervention studies, but none were identified during searching. The included studies do provide insight into the stresses experienced by EDC operatives, but findings are limited by a lack of data on long-term outcomes.

## Conclusions

EDC operatives report experiencing stress as a result of their work, which appears to be related to negative psychological health outcomes, such as emotional exhaustion and burnout. There is, however, a lack of evidence exploring how EDC work-related stress affects operatives’ psychological health over the longer term. Furthermore, most of the studies reviewed only set out to explore potential negative effects on EDC operatives’ psychological health. It is therefore important for future research to explore the positive aspects of the role that operatives value, and to better understand the factors that may contribute to resilience and good psychological health amongst this group.

Future research should also aim to explore the relationships between EDC-related stressors and long-term psychological health outcomes. If longitudinal studies support the main findings of this review, that EDC employment can negatively affect psychological health, there should also be efforts to develop interventions aimed at minimising the impact of EDC work on the psychological health of EDC operatives.

##  Supplemental Information

10.7717/peerj.3735/supp-1 Supplemental Information 1 PRISMA flowchart

10.7717/peerj.3735/supp-2 Supplemental Information 2 PRISMA checklist

10.7717/peerj.3735/supp-3 Supplemental Information 3 Search strings and strategy

10.7717/peerj.3735/supp-4 Supplemental Information 4 Study assessment scoring matrix

## Additional Information and Declarations

### Data Availability

The following information was supplied regarding data availability: The research in this article did not generate any data or code, as it was a systematic literature review. Full details of the search strategy and study inclusion criteria are included in the manuscript and the Supplemental Information.
