# Supplementary material for: Exploring the psychological health of emergency dispatch centre operatives: a systematic review and narrative synthesis
Source: PeerJ. 2017 Oct 17;5:e3735. doi: 10.7717/peerj.3735 (PMC5649589; doi:10.7717/peerj.3735)
Supplement: Supplemental Information 1 [file peerj-05-3735-s001.docx]

Studies included in quantitative synthesis (meta-analysis)
N/A

Studies included in qualitative synthesis
(n = 16)

Full-text articles assessed for eligibility
(n = 115)

Records screened
(n = 1,516)

Records after duplicates removed
(n = 1,516; duplicates = 914)

Full-text articles excluded
(n = 99)

Records excluded
(n = 1,401)

Additional records identified through other sources
(n = 72)

Records identified through database searching
(n = 2,358)

## Identification

## Eligibility

## Included

## Screening
