# Supplementary material for: Exploring the psychological health of emergency dispatch centre operatives: a systematic review and narrative synthesis
Source: PeerJ. 2017 Oct 17;5:e3735. doi: 10.7717/peerj.3735 (PMC5649589; doi:10.7717/peerj.3735)
Supplement: Supplemental Information 2 [file peerj-05-3735-s002.docx]

| **Section/topic** | **#** | **Checklist item** | **Reported on page #** |
| --- | --- | --- | --- |
| **TITLE** | | |  |
| Title | 1 | Identify the report as a systematic review, meta-analysis, or both.  **Exploring the psychological health of emergency dispatch centre operatives: a systematic review and narrative synthesis** | Title Page |
| **ABSTRACT** | | |  |
| Structured summary | 2 | Provide a structured summary including, as applicable: background; objectives; data sources; study eligibility criteria, participants, and interventions; study appraisal and synthesis methods; results; limitations; conclusions and implications of key findings; systematic review registration number. | 2 |
| **INTRODUCTION** | | |  |
| Rationale | 3 | Describe the rationale for the review in the context of what is already known.  **Example excerpt: To date, literature reviews have focused on the sources and consequences of occupational stress that paramedics and other ambulance personnel experience (Sofianopoulos, Williams and Archer, 2012; Hegg-Deloye et al., 2014; Adriaenssens, De Gucht and Maes, 2015). In contrast, there has been no published review exploring the sources of psychological stress when working in an EDC.** | 3-5 |
| Objectives | 4 | Provide an explicit statement of questions being addressed with reference to participants, interventions, comparisons, outcomes, and study design (PICOS).  **The objectives of this systematic review were therefore to investigate and synthesize available evidence relating to the psychological health of EDC operatives, and to identify key stressors that they experience. The specific review question was: what elements of working in the EDC do operatives identify as influencing their psychological health (e.g. reports of negative symptoms of stress, burnout, and mental health conditions, or reports suggesting strong emotional resilience and positive coping skills)?** | 5 |
| **METHODS** | | |  |
| Protocol and registration | 5 | Indicate if a review protocol exists, if and where it can be accessed (e.g., Web address), and, if available, provide registration information including registration number.  **This review was conducted in line with the PRISMA guidelines (see Supplemental File 1) and was registered on the PROSPERO database (http://www.crd.york.ac.uk/PROSPERO): registration number CRD42014010806.** | 5 |
| Eligibility criteria | 6 | Specify study characteristics (e.g., PICOS, length of follow-up) and report characteristics (e.g., years considered, language, publication status) used as criteria for eligibility, giving rationale.  **Participants: Include emergency (ambulance, fire, police) call-handlers and dispatchers (EDC operatives) working in EDCs. Exclude other emergency responders, such as paramedics, police officers, or fire-fighters.**  **Intervention: Any intervention, where applicable.**  **Comparator: Either another intervention, or no intervention (i.e. usual practice or care), where applicable.**  **Outcome Measures: Any psychological health outcome in relation to working within an EDC.**  **Study Design: Any. To include randomized controlled trials, cohort studies, cross-sectional surveys, and qualitative studies.** | 7 |
| Information sources | 7 | Describe all information sources (e.g., databases with dates of coverage, contact with study authors to identify additional studies) in the search and date last searched.  **Eight electronic databases (Embase, PubMed, Medline, CINAHL, PsycInfo, PsycArticles, The Psychology and Behavioural Sciences Collection, and Google Scholar) were searched by SG and CH during May 2016. The contents of the Journal of Paramedic Practice were also reviewed, but this search did not uncover any additional relevant papers. Full search strings are reported in in the additional information provided online.**  **Titles and abstracts of articles retrieved from databases were screened for eligiblity against the inclusion criteria (see below) by SG and CH to determine the population being studied, outcome measures, and study design. Any studies not excluded during abstact review were then sourced for full-text reading. The reference lists of any articles deemed eligible for inclusion after full-text reading were also reviewed, screened and potentially eligible articles retrieved. This process continued until no new articles were identified.** | 6 |
| Search | 8 | Present full electronic search strategy for at least one database, including any limits used, such that it could be repeated.  **The following strings were combined in all databases:**  **“emergency” OR “paramedics” OR “ambulances” OR “first response” OR “first responders” OR “pre-hospital” OR “prehospital” OR “out-of-hospital” OR “out of hospital” OR “fire” OR “police” OR “999” OR “911” OR "111" OR "NHS Direct"**  **AND**  **“dispatch” OR "control centres" or "control centers" OR “call centres” OR “call centers” OR “services” OR “call handlers” OR “call operators” OR “telephone triage”**  **AND**  **“stress” OR “job strain” OR “job demand” OR “job control” OR “job satisfaction” OR “job dissatisfaction” OR “working environment” OR “effort-reward imbalance” OR “burnout” OR “fatigue” OR “exhaustion” OR “sickness absence” OR “demand-resources” OR “distress” OR "recovery" OR “intention to leave” OR “turnover” OR “well-being” OR “wellbeing” OR “sleep quality” OR “sleep disorder” or “coping”.**  **Following this, a supplementary search of google scholar was conducted, (title only, using the following search string: emergency OR paramedics OR ambulances OR "first response" OR "first responders" OR "pre hospital" OR prehospital OR "out of hospital" OR "out of hospital" OR fire OR police AND dispatch OR call OR control), which returned around 325 results (after excluding patents and citations). These titles were scanned for any potential new papers that had not already been identified. Two additional papers were identified for full-text screening, but were not included in the final review.** | Suppl. File 2 |
| Study selection | 9 | State the process for selecting studies (i.e., screening, eligibility, included in systematic review, and, if applicable, included in the meta-analysis).  **Titles and abstracts of articles retrieved from databases were screened for eligiblity against the inclusion criteria (see below) by SG and CH to determine the population being studied, outcome measures, and study design. Any studies not excluded during abstact review were then sourced for full-text reading. The reference lists of any articles deemed eligible for inclusion after full-text reading were also reviewed, screened and potentially eligible articles retrieved. This process continued until no new articles were identified.** | 6 |
| Data collection process | 10 | Describe method of data extraction from reports (e.g., piloted forms, independently, in duplicate) and any processes for obtaining and confirming data from investigators.  **Data extraction was completed by a team of reviewers (AD, BE, SG, CH, MJ, TQ, MR, AS) working in pairs. Each member of a pair independently reviewed and rated their allocated papers, before agreeing final ratings. Collation of ratings and data extraction was overseen by SG and CH to ensure consistent application of assessment tools by all reviewers. Any discrepancies were discussed between SG and CH and amendments agreed.** | 7 |
| Data items | 11 | List and define all variables for which data were sought (e.g., PICOS, funding sources) and any assumptions and simplifications made.  **Data extracted varied according to study design, but in all cases included information about participants and key findings. For qualitative studies, the analytic method and key themes were summarised. For quantitative studies, primary outcome measures were reported.** | 7-8 |
| Risk of bias in individual studies | 12 | Describe methods used for assessing risk of bias of individual studies (including specification of whether this was done at the study or outcome level), and how this information is to be used in any data synthesis.  **The assessment tools used were adapted from the Critical Appraisal Skills Programme checklists (www.casp-uk), as there are qualitative and quantitative versions to enable appraisal across different study designs. For each study, a set of key questions were considered. If the relevant information was clearly reported, a question was scored as ‘yes’. If there was any doubt, or information was not reported in the article, that question was marked as ‘no’ or ‘can’t tell’. Studies were given an overall rating of ‘strong’, ‘moderate’, or ‘weak’, based on the number of questions scored as ‘yes’; studies had to be scored as ‘yes’ on the majority of questions to be rated as ‘strong’ overall (see additional information online for details, as the criteria differed by study design). Greater emphasis was placed upon studies rated as ‘strong’ or ‘moderate’ overall within the findings of the review (Petticrew and Roberts, 2006).**  **Overall scoring was used to make the quality assessment as follows:**   - **Qualitative studies = 9 questions asked:**   - **Score: 0-4 = Weak / 5-7 = moderate / 8-9 = strong** - **Quantitative (Cross-sectional surveys) = 5 questions asked:**   - **Score: 0-2 = Weak / 3-4 = moderate / 5 = strong** - **Quantitative (Cohort) = 9 questions asked:**   - **Score: 0-4 = Weak / 5-7 = moderate / 8-9 = strong** - **Quantitative (Experimental) = 11 questions (asked):**    - **Score: 0-5 = Weak / 6-8 = moderate / 9-11 = strong**   **Note: these thresholds were set prior to ratings being undertaken.** | 8 & Suppl. File 3 |
| Summary measures | 13 | State the principal summary measures (e.g., risk ratio, difference in means). | N/A |
| Synthesis of results | 14 | Describe the methods of handling data and combining results of studies, if done, including measures of consistency (e.g., I^2^) for each meta-analysis.  **Data synthesis**  **Due to heterogeneity of included studies a narrative synthesis was undertaken, using a qualitative approach based on thematic analysis (Braun and Clarke, 2006). Through discussion between four reviewers (SG, CH, AD and MR) key findings from each study were extracted, coded, and grouped into themes and sub-themes. The goal of thematic analysis is to develop a set of themes that are related, but conceptually distinct, and that together illustrate important aspects of the data being analysed. Applying thematic analysis is an iterative and interpretative process, and the themes presented in this review were developed inductively from the data extracted from the included studies. Evidence from both qualitative and quantitative studies was integrated into the findings.** | 8-9 |

Page 1 of 2

| **Section/topic** | **#** | **Checklist item** | **Reported on page #** |
| --- | --- | --- | --- |
| Risk of bias across studies | 15 | Specify any assessment of risk of bias that may affect the cumulative evidence (e.g., publication bias, selective reporting within studies). | N/A |
| Additional analyses | 16 | Describe methods of additional analyses (e.g., sensitivity or subgroup analyses, meta-regression), if done, indicating which were pre-specified. | N/A |
| **RESULTS** | | |  |
| Study selection | 17 | Give numbers of studies screened, assessed for eligibility, and included in the review, with reasons for exclusions at each stage, ideally with a flow diagram.  **A total of 2,358 articles were identified from electronic searching, of which 914 were removed as duplicates. A review of the reference lists of articles included in the review after full-text reading identified a further 72 articles that might potentially meet the inclusion criteria. Therefore, a total of 1,516 abstracts were retrieved for screening. During abstract review, 1,401 articles were excluded, leaving 115 articles to be assessed through full-text reading. After sourcing these for full-text reading, 99 articles were excluded for the following reasons: sample did not include EDC operatives (n = 71), findings were not related to psychological health or stress (n = 18), documents retrieved were unpublished articles or theses (n = 10). This resulted in 16 articles meeting the inclusion criteria.** | 9 |
| Study characteristics | 18 | For each study, present characteristics for which data were extracted (e.g., study size, PICOS, follow-up period) and provide the citations.  **Refer to section ‘Study Characteristics’** | See pages 9-11 & Tables 3-6 |
| Risk of bias within studies | 19 | Present data on risk of bias of each study and, if available, any outcome level assessment (see item 12).  **Refer to section ‘Risk of Bias within Studies’** | See page 10 & Tables 1-2 |
| Results of individual studies | 20 | For all outcomes considered (benefits or harms), present, for each study: (a) simple summary data for each intervention group (b) effect estimates and confidence intervals, ideally with a forest plot. | N/A |
| Synthesis of results | 21 | "Present the main results of the review. If meta-analyses are done, include for each, confidence intervals and measures of consistency"  **Refer to section ‘Narrative Synthesis’** | See pages 11 - 20 |
| Risk of bias across studies | 22 | Present results of any assessment of risk of bias across studies (see Item 15). | N/A |
| Additional analysis | 23 | Give results of additional analyses, if done (e.g., sensitivity or subgroup analyses, meta-regression [see Item 16]). | N/A |
| **DISCUSSION** | | |  |
| Summary of evidence | 24 | Summarize the main findings including the strength of evidence for each main outcome; consider their relevance to key groups (e.g., healthcare providers, users, and policy makers).  **Opening paragraph of discussion: Research examining Emergency Dispatch Centre (EDC) operatives’ psychological health is limited. This narrative review identified that EDC operatives across different emergency services consistently reported their job as highly stressful, and this stress sometimes affected their psychological health. It also highlighted a lack of longitudinal studies exploring the long-term effects on psychological health of working in an EDC.**  **Closing paragraph of discussion: The findings from this review therefore highlight the unique combination of challenges faced by EDC operatives. The role combines features of both regular call-centres and emergency settings. Operatives report facing a psychologically demanding work environment, and at times, the workload can seem relentless. Although some operatives appear to thrive on the challenge, a significant proportion of the EDC workforce report experiencing negative effects on their psychological health as a result of their work. Future research should focus on exploring whether workplace-based interventions can improve the psychological health of those EDC operatives who are negatively affected.** | 21 - 24 |
| Limitations | 25 | Discuss limitations at study and outcome level (e.g., risk of bias), and at review-level (e.g., incomplete retrieval of identified research, reporting bias).  **Search strings were devised to capture a broad range of studies, and a number of common themes were identified. The diversity of EDC populations studied (across time, role, and location) means the relative importance of each theme is likely to vary between each sub-population of EDC operatives; there were, however, insufficient publications to enable differences between the emergency services to be explored. As there is a lack of recent evidence in particular, the findings of the review are potentially both time and context-bound. Results should be interpreted with caution as findings are based on a limited number of high quality studies and there was heterogeneity in study design. Nonetheless, there was evidence of consistency across studies, as the themes identified were supported by evidence from all three emergency services, suggesting that the findings of the review are likely to be relevant to all types of EDC operatives.**  **The review was intended to capture longitudinal and intervention studies, but none were identified during searching. The included studies do provide insight into the stresses experienced by EDC operatives, but findings are limited by a lack of data on long-term outcomes.** | 24-25 |
| Conclusions | 26 | Provide a general interpretation of the results in the context of other evidence, and implications for future research.  **EDC operatives report experiencing stress as a result of their work, which appears to be related to negative psychological health outcomes, such as emotional exhaustion and burnout. There is, however, a lack of evidence exploring how EDC work-related stress affects operatives’ psychological health over the longer term. Furthermore, most of the studies reviewed only set out to explore potential negative effects on EDC operatives’ psychological health. It is therefore important for future research to explore the positive aspects of the role that operatives value, and to better understand the factors that may contribute to resilience and good psychological health amongst this group.**  **Future research should also aim to explore the relationships between EDC-related stressors and long-term psychological health outcomes. If longitudinal studies support the main findings of this review, that EDC employment can negatively affect psychological health, there should also be efforts to develop interventions aimed at minimising the impact of EDC work on the psychological health of EDC operatives.** | 25 |
| **FUNDING** | | |  |
| Funding | 27 | Describe sources of funding for the systematic review and other support (e.g., supply of data); role of funders for the systematic review. | Title Page |

*From:*  Moher D, Liberati A, Tetzlaff J, Altman DG, The PRISMA Group (2009). Preferred Reporting Items for Systematic Reviews and Meta-Analyses: The PRISMA Statement. PLoS Med 6(6): e1000097. doi:10.1371/journal.pmed1000097

For more information, visit: **www.prisma-statement.org**.

Page 2 of 2
