# Supplementary material for: Exploring the psychological health of emergency dispatch centre operatives: a systematic review and narrative synthesis
Source: PeerJ. 2017 Oct 17;5:e3735. doi: 10.7717/peerj.3735 (PMC5649589; doi:10.7717/peerj.3735)
Supplement: Supplemental Information 3 [file peerj-05-3735-s003.docx]

**APPENDIX A**

**Search Strings and Strategy Details**

The following strings were combined in all databases:

“emergency” OR “paramedics” OR “ambulances” OR “first response” OR “first responders” OR “pre-hospital” OR “prehospital” OR “out-of-hospital” OR “out of hospital” OR “fire” OR “police” OR “999” OR “911” OR "111" OR "NHS Direct"

AND

“dispatch” OR "control centres" or "control centers" OR “call centres” OR “call centers” OR “services” OR “call handlers” OR “call operators” OR “telephone triage”

AND

“stress” OR “job strain” OR “job demand” OR “job control” OR “job satisfaction” OR “job dissatisfaction” OR “working environment” OR “effort-reward imbalance” OR “burnout” OR “fatigue” OR “exhaustion” OR “sickness absence” OR “demand-resources” OR “distress” OR "recovery" OR “intention to leave” OR “turnover” OR “well-being” OR “wellbeing” OR “sleep quality” OR “sleep disorder” or “coping”.

Following this, a supplementary search of google scholar was conducted, (title only, using the following search string: emergency OR paramedics OR ambulances OR "first response" OR "first responders" OR "pre hospital" OR prehospital OR "out of hospital" OR "out of hospital" OR fire OR police AND dispatch OR call OR control), which returned around 325 results (after excluding patents and citations). These titles were scanned for any potential new papers that had not already been identified. Two additional papers were identified for full-text screening, but were not included in the final review.
