# Supplementary material for: Exploring the psychological health of emergency dispatch centre operatives: a systematic review and narrative synthesis
Source: PeerJ. 2017 Oct 17;5:e3735. doi: 10.7717/peerj.3735 (PMC5649589; doi:10.7717/peerj.3735)
Supplement: Supplemental Information 4 [file peerj-05-3735-s004.docx]

**APPENDIX B**

**Scoring Matrix for Quality Assessment**

Overall scoring was used to make the quality assessment as follows:

- Qualitative studies = 9 questions asked:
  - Score: 0-4 = Weak / 5-7 = moderate / 8-9 = strong
- Quantitative (Cross-sectional surveys) = 5 questions asked:
  - Score: 0-2 = Weak / 3-4 = moderate / 5 = strong
- Quantitative (Cohort) = 9 questions asked:
  - Score: 0-4 = Weak / 5-7 = moderate / 8-9 = strong
- Quantitative (Experimental) = 11 questions (asked):
  - Score: 0-5 = Weak / 6-8 = moderate / 9-11 = strong

Note: these thresholds were set prior to ratings being undertaken.
